# Bimetallic MOF-based electrochemical sensor for determination of paracetamol in spiked human plasma

**DOI:** 10.1186/s13065-024-01247-7

**Published:** 2024-08-08

**Authors:** Aya A. Mouhamed, Ahmed H. Nadim, Amr M. Mahmoud, Nadia M. Mostafa, Basma M. Eltanany

**Affiliations:** Pharmaceutical Analytical Chemistry Department, Faculty of Pharmacy, Cairo University, Kasr El-Aini St, Cairo, 11562 Egypt

**Keywords:** Bimetallic metal-organic framework, Monometallic metal-organic framework, Carbon paste electrode, Electrochemical sensor, Paracetamol, *p*-aminophenol

## Abstract

Metal-organic frameworks (MOFs) with their exceptional properties have the potential to revolutionize the field of electrochemistry and pave the way for new and exciting applications. MOFs is an excellent choice as an active electrocatalyst component in the fabrication of electrochemical sensors. Here, bimetallic NiCo-MOFs, monometallic Ni-MOFs, and Co-MOFs were fabricated to modify the carbon paste electrode. Moreover, the ratio between Co and Ni within the bimetallic MOFs was optimized. Our aim in this work is to synthesize different compositions from bimetallic MOFs and systematically compare their catalytic activity with mono-metallic MOFs on paracetamol. The structure and properties of the 2D NiCo-MOFs were characterized by scanning electron microscope, X-ray photoelectron spectroscopy, Fourier transform infrared, and electrochemical method. Bimetallic Ni_0.75_Co_0.25_-MOFs modified carbon paste sensor displayed the optimum sensing performance for the electrochemical detection of paracetamol. A linear response over the range 6.00 × 10^− 7^ to 1.00 × 10^− 4^ M with a detection limit of 2.10 × 10^− 8^ M was obtained. The proposed method was applied to detect paracetamol in spiked human plasma and to determine paracetamol in the presence of its major toxic impurity, *p*-aminophenol. These findings suggest the considerable potential use of the newly developed sensor as a point-of-care tool for detecting paracetamol and *p*-aminophenol in the future.

**Supplementary Information:**

The online version contains supplementary material available at 10.1186/s13065-024-01247-7.

## Introduction

Electrochemical sensors are a remarkable class of sensors in which the electrode is the transducer element. These sensors (which appeared in the second half of the twentieth century) are now found in a wide range of commercial applications [1]. These sensors use the electrons to acquire signals, which is considered a clean model for analytical applications, with no generation of waste. In addition, they are miniaturized for use in portable devices, which allows for analyses with micro volumes of samples [1, 2]. Electrocatalysis is crucial for modern energy storage and conversion [3]. Advanced electrocatalysts help to decrease energy consumption in electrochemical processes [4]. Metal-organic frameworks (MOFs) which were reported in 1995 by Yaghi et al. [5], have shown great promise as electrocatalysts in recent years [4]. They represent a class of solid porous materials, which consist of metal nodes, and polydentate organic linkers [6]. They are considered a subset of coordination polymers and can be extended into one, two, and three dimensions [7]. MOFs have a wide diversity of skeleton and pores, network topology, surface functionalities, and charge mobility that result in tunable porosity [8]. They also introduce exciting new avenues for creativity in designing ligands, assembling crystal engineering [9], chemical separations, photocatalysis [10, 11], electrocatalysis [12], and energy storage materials candidates [7, 13]. Several strategies were developed to enhance the catalytic activity, such as element doping [14, 15], coordination structures optimization, and or defects construction [16–18]. Another strategy is the regulation of the number of accessible active sites of MOFs by different methods such as morphology engineering [19–21]. Unlike monometallic compounds, bimetallic MOFs provide numerous benefits for use as electrode components or as precursors in the production of advanced composites due to higher stability and efficiency [22]. Many experimental results proved that the inclusion of cobalt (Co) and nickel (Ni) transition elements creates more electrochemically active sites and increases catalytic activity [23, 24]. In 2016, Zhao et al. published a study of the electrocatalytic performance of NiCo bimetal-organic framework nanosheets on the oxygen evolution reaction [25].

**Fig. 1:**
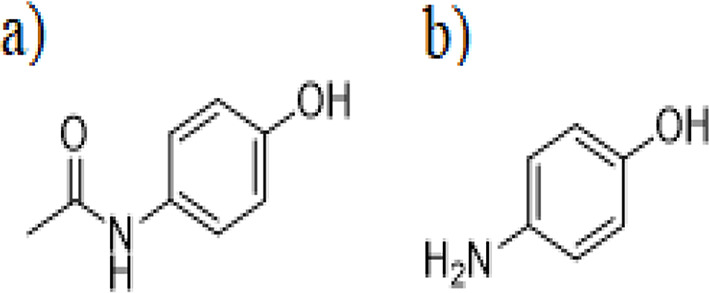
Chemical structure of PAR and *p*-AP

Paracetamol (PAR), N-(4-hydroxyphenyl) acetamide (Fig. 1a) is commonly used as a painkiller and antipyretic. It is a suitable alternative for aspirin-sensitive patients [26]. PAR is usually used to relieve moderate pain, such as headaches caused by the flu, joint pain, and migraines [27]. The normal dose of PAR is not harmful to the human body [28]. However, PAR is a substrate of oxidative metabolism by Cytochrome P450 2E1 (CYP2E1), generating the toxic metabolite N-acetyl-p-benzoquinone imine (NAPQI). Overdosage of PAR can lead to hepatic necrosis through the diminution of cellular glutathione levels by forcing NAPQI to interact with nucleophilic cellular macromolecules [28]. The widespread availability and usage of PAR increase the incidence of accidental or intentional overdoses globally [29]. Each year, PAR overdoses result in approximately 56,000 emergency room visits, 33,000 hospitalizations, and 500 deaths [29].

Like many other drugs, PAR undergoes degradation during storage. The manufacturing process also introduces some impurities [30]. Para-aminophenol (*p*-AP), (Fig. 1b) is the primary PAR impurity. It is nephrotoxic and can cause teratogenicity [31].

Therefore, a simple, sensitive, and precise method of determining PAR in the presence of *p*-AP is significant for personal health care. Compared to traditional spectrophotometry [32]or chromatography [33], the electrochemistry technique is simple, has a fast response, high sensitivity, and low cost [34–36]. The detection of PAR by electrochemical sensors has attracted much interest in recent years. Various designs were detected including screen-printed electrodes [37], carbon-based electrodes [38], and nanomaterial-modified electrodes [39], to enhance sensitivity and selectivity towards PAR. The choice of electrode materials is critical for the sensor’s performance. Carbon-based materials such as graphene, carbon nanotubes, and carbon nanofibers have been widely investigated due to their high conductivity, large surface area, and electrocatalytic properties [40]. Surface functionalization techniques, such as electrodeposition, molecular imprinting [38], and electropolymerization [38], have been employed to enhance the selectivity of electrochemical sensors toward PAR. Electrochemical detection methods for PAR include voltammetric techniques, such as cyclic voltammetry (CV) and differential pulse voltammetry (DPV), which are commonly used for their high sensitivity and ease of implementation in sensor devices [41]. Efforts have been made to miniaturize electrochemical sensors for PAR detection, to be integrated into portable devices for on-site analysis [42]. Several strategies have been developed to enhance the selectivity of electrochemical sensors for PAR detection by minimizing interference from other electroactive species found in real samples [42]. This includes the use of selective electrode materials, signal processing, and sample pretreatment techniques [43, 44].

In our presented study, novel functional nanomaterials have been developed to create modified electrodes for the sensitive and specific electrochemical detection of PAR in the presence of its primary impurity, p-AP. Bimetallic NiCo-metal-organic frameworks (NiCo-MOFs) were used to modify carbon paste electrodes (CPE), which were synthesized and subsequently compared for catalytic activity with monometallic Ni-MOFs/CPE and Co-MOFs/CPE using PAR as a representative drug model. Results revealed that NiCo-MOFs/CPE exhibited significant electrocatalytic performance in the redox process of PAR compared to both monometallic Ni-MOFs/CPE and Co-MOFs/CPE. Furthermore, the developed sensor was successfully employed for PAR detection in pharmaceutical formulations and spiked human plasma samples.

MOFs-modified sensors can maintain their sensing performance over extended periods due to their known structure stability and this ensures reliable and continuous monitoring of PAR levels without significant degradation. MOFs-modified sensors can be integrated into portable and miniaturized devices for on-site detection of PAR. This has implications in point-of-care diagnostics, where rapid and sensitive detection of paracetamol levels is crucial for patient care.

## Experimental

### Materials and reagents

Polyvinyl pyrrolidine K30 (PVP), purified terephthalic acid (PTA), nickel (II) sulfate hexahydrate, cobalt (II) chloride hexahydrate, dimethyl formamide (DMF), sodium hydroxide, graphite, phosphate-buffered saline (PBS) tablets, and p-aminophenol were purchased from Sigma-Aldrich. PAR was obtained from the Egyptian Drug Authority (EDA) (with a certified purity of 99.58% ± 1.01). Ethanol of HPLC grade was supplied from Fisher Scientific (Loughborough, Leicestershire, UK). Deionized water was prepared by double distillation (Agela Technologies Wilmington, USA). Blank human plasma was supplied from the Holding Company for Biological Products and Vaccines (VACSERA, Egypt).

### Instrumentation

A PC-controlled electrochemical analytical workstation (Metrohm Autolab potentiostat/ galvanostat PGSTAT204) supplied with NOVA software for electrochemistry was applied for all voltammetric measurements. The used reference electrode was Ag/AgCl, while a Pt wire was utilized as a counter electrode. The working electrode used was CPE. For the analysis of spiked human plasma, the samples were mixed using a vortex (VELP Scientifica, Europe), and then centrifuged using a Centurion K241R centrifuge (UK). The solution’s pH was adjusted using Jenway digital ion analyzer model 3330 with Jenway pH glass electrode (Essex, UK). The SEM image of Ni_0.75_: Co_0.25_-MOFs was obtained using the ZEISS EVO scanning electron microscope in Hamburg, Germany. The structure of Ni_0.75_: Co_0.25_-MOFs was characterized through XPS, K-ALPHA (Thermo Fisher Scientific, USA) utilizing monochromatic X-ray Al K-alpha radiation − 10 to 1350 eV, spot size 400 μm, at specified pressure 10^−9^ mbar with full spectrum pass energy 200 eV and narrow spectrum at 50 eV. Fourier transform infrared spectrum (FT-IR) of the NiCo- MOFs was obtained by an FTS- 3000 spectrometer in the range from 4000 to 400 cm^− 1^.

### Fabrication of MOFs

#### Fabrication of monometallic Ni-MOFs and Co-MOFs

A solvothermal method was used to prepare Ni-MOFs [12]. Thus, NiSO_4_.6H_2_O (0.144 g), PTA (0.25 gm), and PVP (0.5 gm) were poured in DMF (30 mL), then 3mL 0.4 M NaOH was added dropwise while stirring for 20.0 min. A faint green colloidal suspension was obtained and then subjected to a solvothermal reaction for 8 h at 100 °C inside a 50-mL Teflon autoclave. Next, the product was cooled down to room temperature, followed by centrifugation at 4000 rpm for 14.0 min to obtain the precipitate then washed with DMF and ethanol, and finally dried at 65 °C for 1 h under vacuum condition. Moreover, Co-MOFs was prepared by the same procedure using CoCl_2_.6H_2_O.

#### Fabrication of bimetallic Ni/Co-MOFs

Different molar ratios of Ni and Co were prepared (1:1), (3:1), and (1:3) then the same procedures were applied to prepare different bimetallic NiCo-MOFs as mentioned in.

### Fabrication of MOFs modified carbon paste electrodes

The working modified electrodes were prepared as follows: 190.00 mg graphite was weighed and placed in mortar. Then 10.0 mg of synthesized Ni-MOFs, Co-MOFs, Ni_0.25_Co_0.75−_MOFs, Ni_0.5_Co_0.5−_MOFs, and Ni_0.75_Co_0.25−_MOFs mixed at least for 5.0 min using 0.50 mL paraffin oil. Each modified paste was filled in the electrode body and a copper wire was used for electrode connection. The CPE was prepared in the same procedure without MOFs mixing. The new surface of the paste electrode surface was simply renewed by scraping off about 3.0 mm of its old surface and polishing the new surface with a piece of tracing paper. Modified CPE sensors with varied MOFs composition can be recorded as Ni-MOFs/CPE, Co-MOFs/CPE, Ni_0.25_Co_0.75_-MOFs/CPE, Ni_0.5_Co_0.5_ -MOFs/CPE, and Ni_0.75_Co_0.25_ -MOFs/CPE.

### Procedure

#### Standard solution

Stock standard solution of PAR was prepared by accurately weighing 0.38 g of pure drug and transferring it to a 100-mL volumetric flask then dissolved in 50-mL distilled water and completed to the mark with distilled water. Then one mL from the prepared solution was transferred into a 25-mL volumetric flask and completed to the mark with distilled water to obtain a PAR solution of concentration equal to 10^–3^M.

#### Operational conditions of electrochemical measurements

Cyclic wave voltammograms were obtained by scanning the potential over a range of -0.1 to 1.2 V, starting from 0. A carbon paste electrode (CPE) was used versus the reference electrode Ag/AgCl, with a platinum wire as a counter electrode. All determinations were carried out at room temperature. DPV measurements were conducted by scanning the potential over a range of -0.4 to 1.0 V at a scan rate of 40 mV/s. The sample width, modulation amplitude, pulse width (modulation time), pulse period (interval), and quiet time were set to 17.0 ms, 40.0 mV, 50.0 ms, 600.0 ms, and 5.0 s, respectively. These parameters were carefully chosen to ensure accurate and reliable measurements.

#### Construction of calibration curve

Aliquots of PAR were transferred from the stock solution to 25-ml volumetric flasks. The volumes were then accurately completed using PBS pH 7.4 to produce the desired final concentrations ranging from 0.60 µM to 100.00 µM. DPV was produced between -0.4 and 1.0 V. The current peak height confidently recorded for each sample was plotted against its corresponding concentration to determine the calibration curve and regression equation.

### Method validation

The developed method was successfully validated following ICH guidelines [45] for linearity, accuracy, precision, detection, and quantification limits, confirming its suitability for the intended use. Linearity was thoroughly investigated under optimal electrochemical conditions by analyzing six different concentrations of PAR. Accuracy was rigorously assessed by calculating the percentage recovery of three different concentrations. Intra-day and inter-day precision were evaluated and reported as %RSD.

### Application procedure

#### *Analysis of PAR in real samples*

For the preparation of the real sample of PAR tablet (labelled 500 mg), ten PAR tablets were crushed. Then, 5.09 mg of this powder was dissolved in 25.00 mL of deionized water under ultra-sonication to prepare a concentration of 10^− 3^ M. The resulting sample was filtered using filter paper to remove impurities and fillers in the tablet.

A specific volume of this solution was transferred to a volumetric flask (25 mL) and diluted with PBS (pH = 7.4). Finally, the obtained concentrations were analyzed to calculate the recovery percentage.

#### Analysis of PAR in spiked human plasma

A standard stock solution of 1.00 mM PAR was prepared by dissolving 3.80 mg of pure PAR powder in 25.00 mL of distilled water. From this solution, various working standard solutions were prepared. Then, 500.00 µL was withdrawn from each working solution and added separately to 500.00 µL of plasma in a 10-mL centrifuge tube. Protein precipitation was efficiently achieved by adding 1.50 mL of acetonitrile. The mixtures were blended using a vortex mixer for 5.0 min at 4000 rpm, followed by a carefully timed centrifugation for 15.0 min at 4000 rpm. The supernatant was transferred and evaporated to complete dryness utilizing the rotatory vacuum concentrator at 60. Samples were reconstituted in 25.00 mL of PBS to obtain the final desired concentrations of 1.00 × 10^–5^, 4.00 × 10^–5^, 6.00 × 10^–5^, 8.00 × 10^–5^, and 1.00 × 10^–4^ M. DPV was generated for those spiked samples in the range from -0.4 to 1.0 V. To construct a calibration curve, the current peak heights of the measured concentrations were used and correlated with their corresponding concentrations. To evaluate the accuracy, the percentage recovery of three different concentrations was calculated.

## Results and discussion

**Fig. 2:**
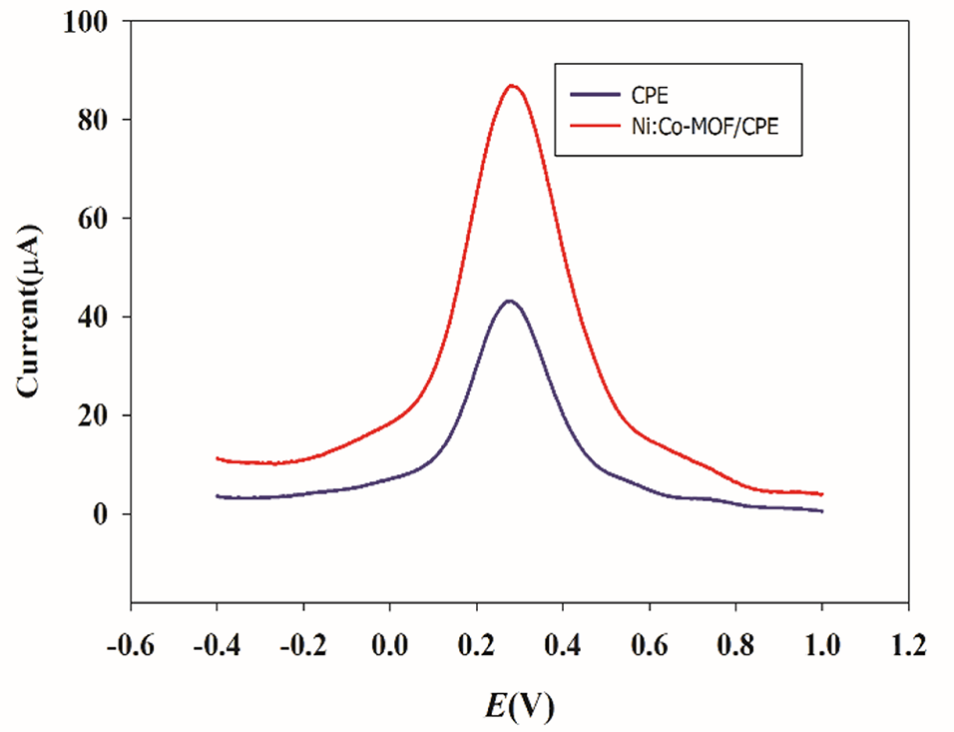
Differential pulse voltammograms of PAR using potassium ferro/ferricyanide solution (0.01 mM) in PBS buffer at solid CPE with/without NiCo-MOFs

**Fig. 3:**
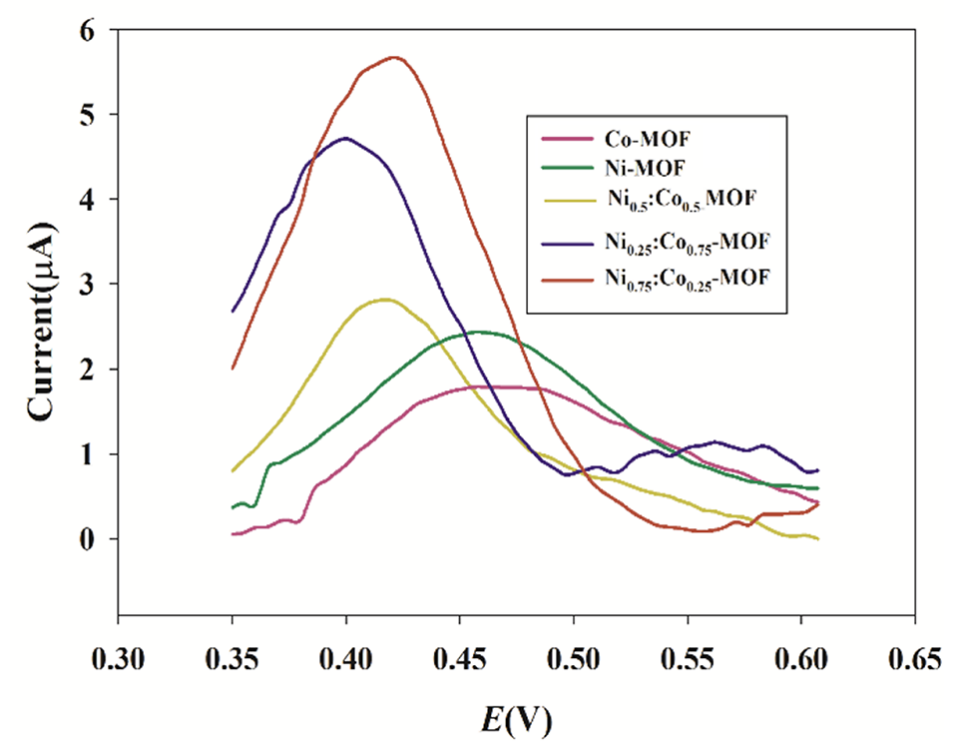
Differential pulse voltammograms of PAR on the Ni-MOFs/CPE, Co-MOFs/CPE, Ni_0.25_Co_0.75_-MOFs/CPE, Ni_0.5_Co_0.5_-MOFs/CPE, and Ni_0.75_Co_0.25_-MOFs/CPE using scan rate 40 mV/s and pH 7.4

**Fig. 4:**
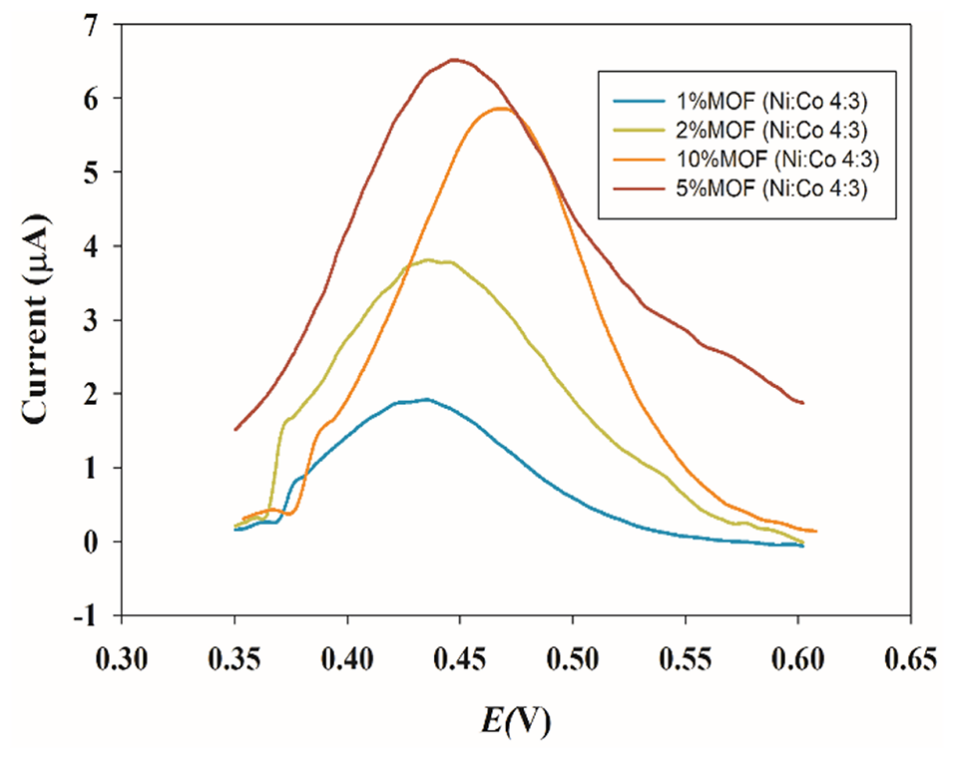
Differential pulse voltammograms of PAR on different percentages of Ni_0.75_Co_0.25_-MOFs (1%, 2%, 5%,10%) using scan rate 40 mV/s and pH 7.4

Compared to traditional porous materials, the most attractive advantage of MOFs is that their catalytic activity can be enhanced using different metal ions and organic linkers. Purified terephthalic acid (PTA) was used as a ligand. Nickel sulfate and cobalt chloride were used as ion sources to synthesize 2D MOFs through a solvothermal method [12]. PVP was assembled as a polymer in the fabrication of MOFs to prevent aggregation and provide a larger surface area and more active sites to interact with the PAR. To investigate the redox property of the CPE sensor modified with the porous NiCo-MOFs, a DPV study was conducted on plain CPE and NiCo-MOFs/CPE sensors as shown in Fig. 2. The NiCo-MOFs/CPE sensor exhibited better-defined oxidation and reduction properties of the ferro/ferri cyanide solution than the plain CPE sensor. The synthesized bimetallic NiCo-MOFs have more catalytic activity than monometallic Ni-MOFs and Co-MOFs, Fig.  3 and Figure S1. The catalytic performance of different molar ratios of Ni and Co were examined (Ni_0.25_Co_0.75_, Ni_0.5_Co_0.5_, Ni_0.75_Co_0.25_) and compared, results are shown in Fig. 3. The molar ratio of Ni_0.75_Co_0.25_-MOFs was revealed to have the most catalytic activity, Fig. 3. Then several percentages of the optimized molar ratio Ni_0.75_Co_0.25_-MOFs (1%, 2%, 5%, and 10%) were carried out. The best results were achieved by applying 5% Ni_0.75_Co_0.25_-MOFs to CPE where a high current was obtained, Fig.  4.

### Surface characterization of bimetallic Ni_0.75_Co_0.25_-MOFs/CPE

**Fig. 5:**
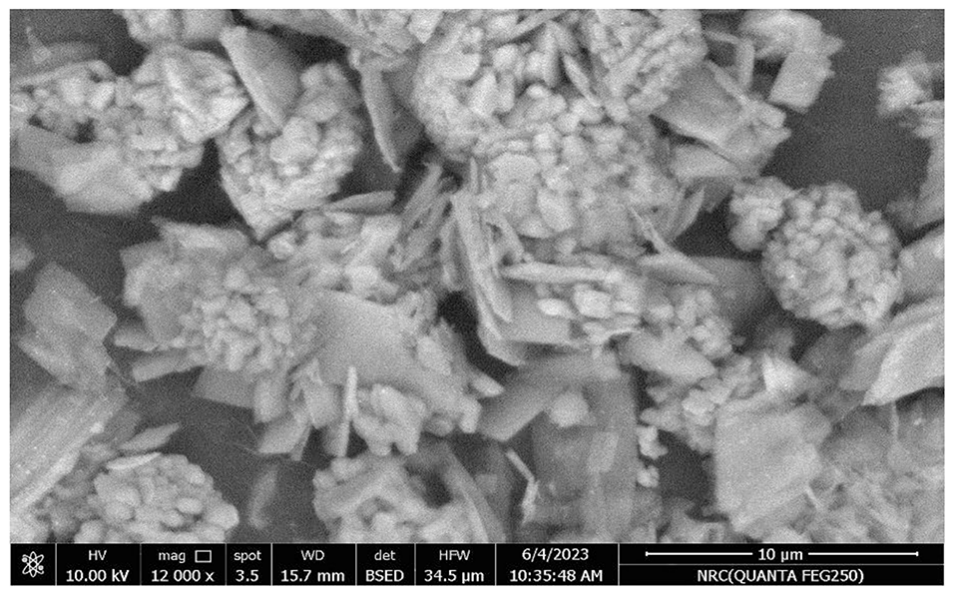
SEM image of cauliflower-like Ni_0.75_Co_0.25_-MOFs

**Fig. 6:**
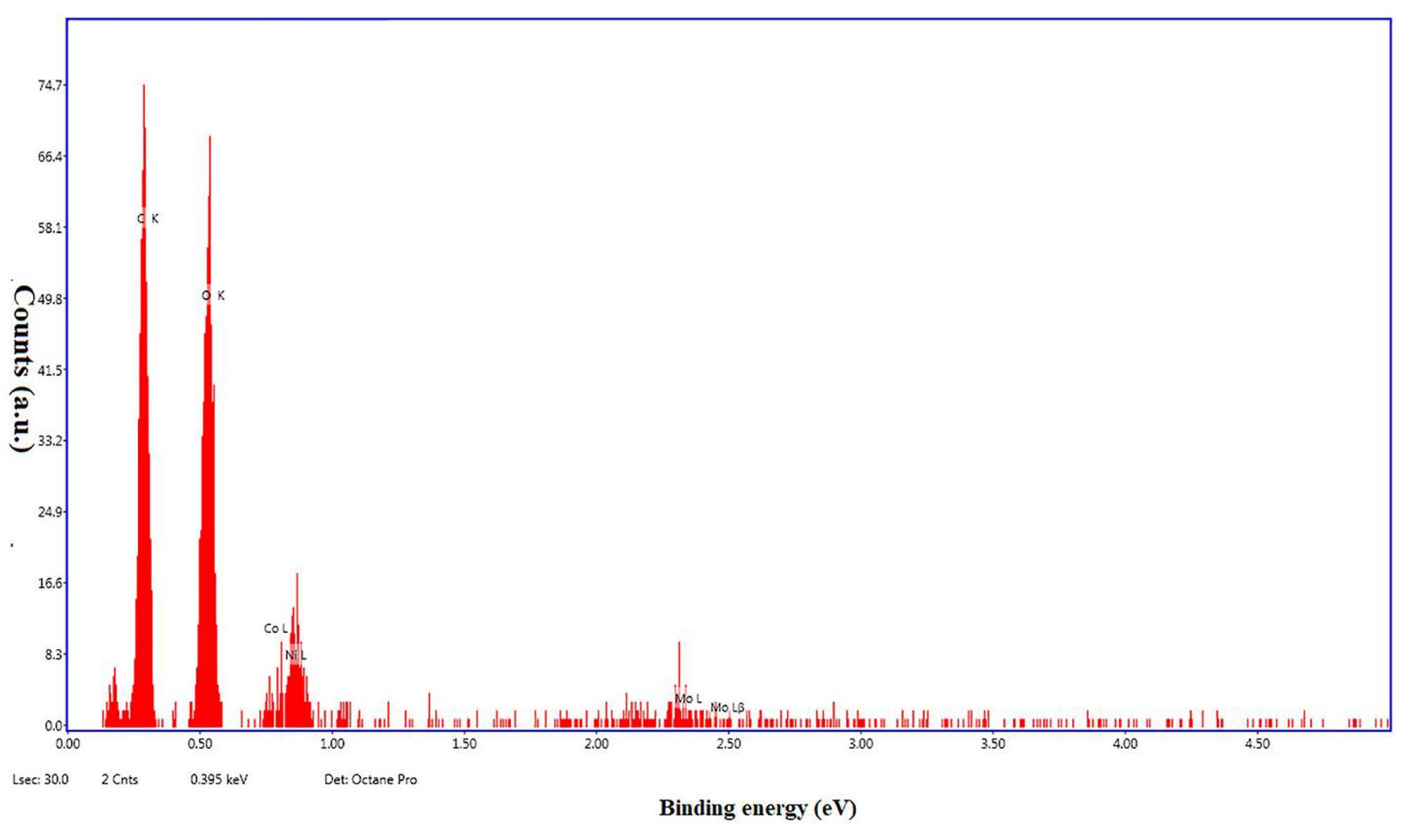
EDX spectrum of Ni_0.75_ Co_0.25_-MOFs

The morphology of the synthesized NiCo-MOFs was examined using SEM. A cauliflower-shaped structure aggregates were obtained with average length of 5.4 ± 1.3 μm and width of 3.1 ± 0.74 μm of the cluster Fig.  5. Moreover, energy dispersion X-ray (EDX) analysis was used for the elemental analysis. The elemental mapping of NiCo-MOFs confirmed the presence of Ni, Co, C, and O in the structure Fig. 6. This was also confirmed by the XPS technique that was used to analyze the elemental structure of NiCo-MOFs. The survey spectrum clearly showed the presence of Ni, Co, O, N, and C elements. The high-resolution XPS spectra were obtained. The Co(2p) high-resolution XPS spectrum shows three peaks at 780.4, 785.3, and 797.0 eV corresponding to Co^2+^ oxidation state. Similarly, Ni(2p) high-resolution XPS spectrum showed peaks at 855.6, 861.1, and 873.6 eV that could be assigned to Ni^2+^ oxidation state. Such results showed electronic interactions between Co and Ni atoms found in Ni_0.75_Co_0.25_-MOFs which would improve the electro-catalytic properties of MOFs. The C(1s) spectra showed peaks at around 283.9, 284.1, and 287.6 eV corresponding to C = C, C = O, and O-C = O. The N(1s) spectra had two major peaks at 399.58 eV for pyridinic-N and 401.1 eV for pyrrolic-N indicating the formation of MOFs on the PVP polymer. From the infrared spectrum of Ni_0.75_Co_0.25−_MOFs (Figure. S2), it can be observed that a new characteristic absorption peak appears at 1377 cm^− 1^ and 1577 cm^− 1^ which corresponded to asymmetric and symmetric stretching modes of coordinated carboxylic acid, respectively [46]while the broad peaks in the spectral range of 3150–3400 cm^− 1^ may be due to acidic OH of carboxylic groups [47]. The peaks at 1161 and 1099 m^− 1^ are attributed to the in-plane stretching vibration of C-H while the peaks at 756 and 813 cm^− 1^ are assigned to the out-of-plane bending vibration of C-H [48]. The absorptions around 466, 524, 559, and 609 cm^− 1^ are related to metal–oxygen–hydrogen bending vibration (Ni-O or Co-O or Ni-Co-O bond) [47].

### Electrochemical characterization

Electrochemical Impedance Spectroscopy (EIS) of different electrodes was often performed using [Fe (CN)6]^−3^/ [Fe (CN)6]^−4^ redox probes [49–51]. As shown in Figure S3, the different sizes of the semicircle for each curve, it can be concluded that the Charge transfer resistance *(Rct)* of Ni _0.75_Co_0.25_-MOFs/CPE was significantly lower than that of CPE. The *Rct* of CPE and Ni _0.75_Co_0.25_-MOFs/CPE were 1.15, 0.788 kΩ. This suggests that Ni _0.75_Co_0.25_-MOFs was successfully coated on the electrode surface, and the introduction of conductive Ni _0.75_Co_0.25_-MOFs was a reliable way to reduce the *Rct* of CPE and thus promote electron transfer.

### Electrochemical performance of PAR on the Ni_0.75_Co_0.25_-MOFs/CPE surface

#### The effect of buffer and pH

**Fig. 7:**
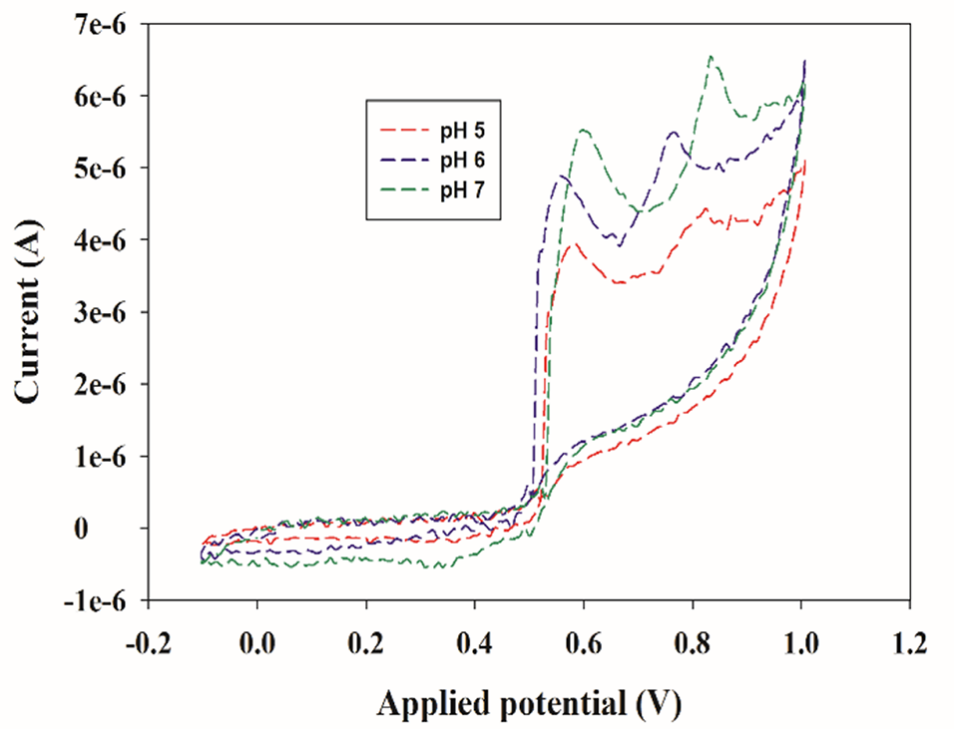
Effect of pH on the cathodic peak current (Ip)

To obtain better electrochemical sensing performance, the detection conditions were optimized using CV. Firstly, three types of buffers were tried: acetate, Britton -Robinson Buffer (BRB), and PBS each at pH 7.0. The peak potential of PAR was maximum with the PBS buffer. Then, the pH ranges between 5.0 and 7.0 through PBS buffer were studied to determine the impact of the pH value of the electrolyte on the response of PAR. Because the pKa value is 9.5 for PAR, therefore it carries a positive charge at pH values lower than their pKa values [52]. As shown in Fig. 7, the oxidation peak current of PAR increased as pH values increased from 5.0 to 7.0 as at lower pH, PAR is more protonated, meaning it has gained a hydrogen ion (H^+^) and exists in a charged form. This may hinder its oxidation process, resulting in lower peak currents. As the pH increases to pH 7.0 (Neutral), PAR becomes less protonated and more in its neutral form. This neutral form enhanced electrochemical activity, leading to higher oxidation peak currents [53]. For achieving higher sensitivity, pH 7.4 was selected as an optimal pH to electrochemically detect PAR on the Ni_0.75_Co_0.25_-MOFs/CPE.

#### The effect of scanning rate

**Fig. 8:**
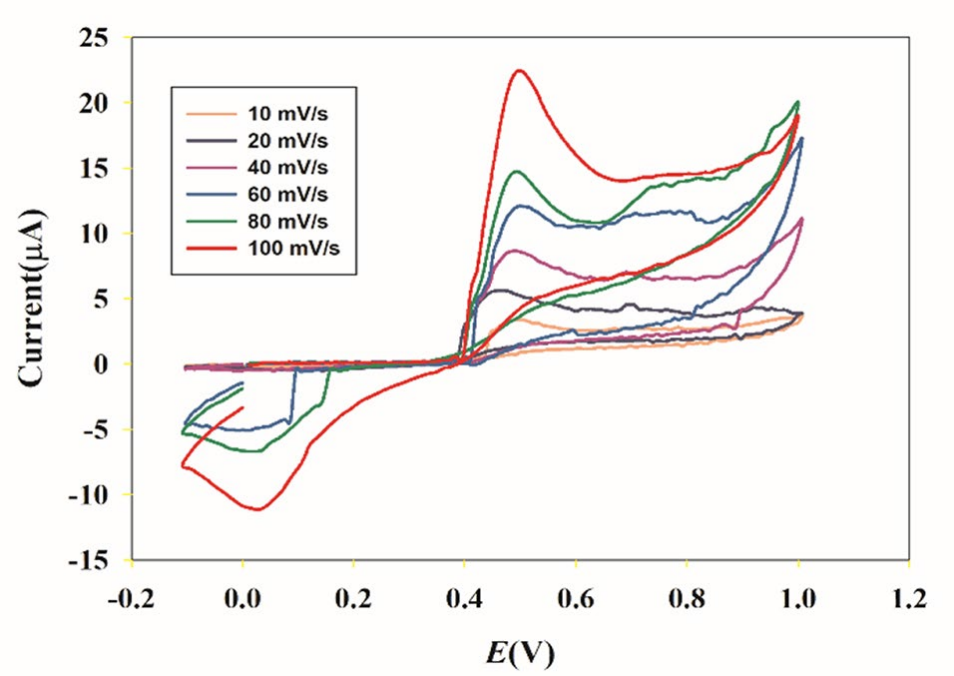
Cyclic Voltammograms of 0.1mM PAR in 10 mM PBS (pH 7.4) on the Ni_0.75_Co_0.25_-MOFs /CPE at different scanning rates: 10, 20, 40, 60, 80, and 100 mV/s

**Fig. 9:**
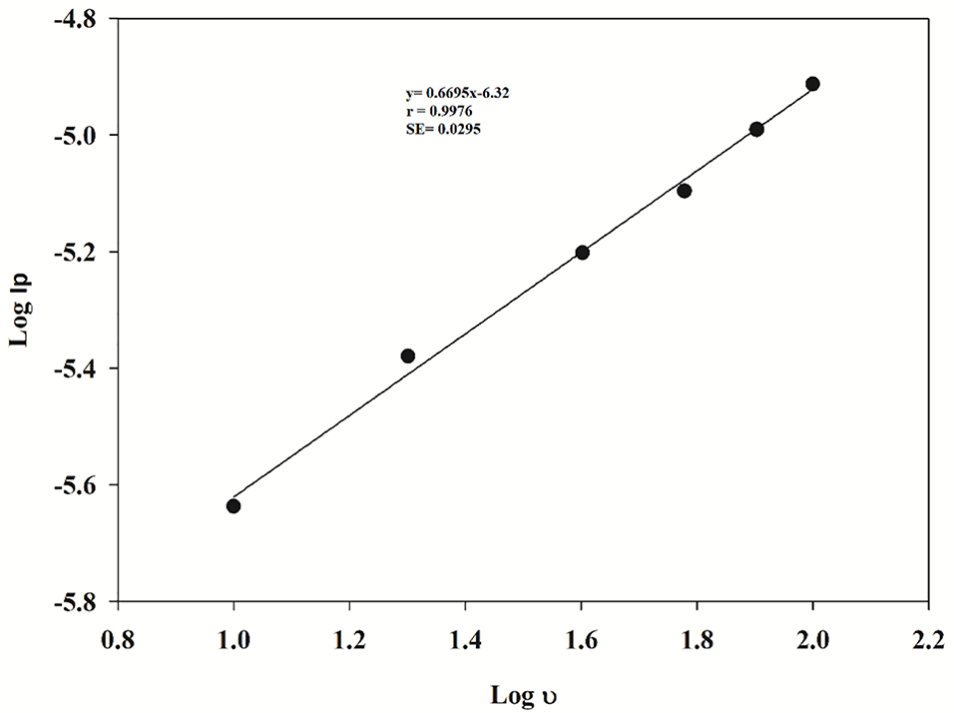
A plot of logarithm of anodic peak current (log Ip) of PAR as a function of the logarithm of scan rate (log ʋ) using cyclic voltammetry

**Fig. 10:**
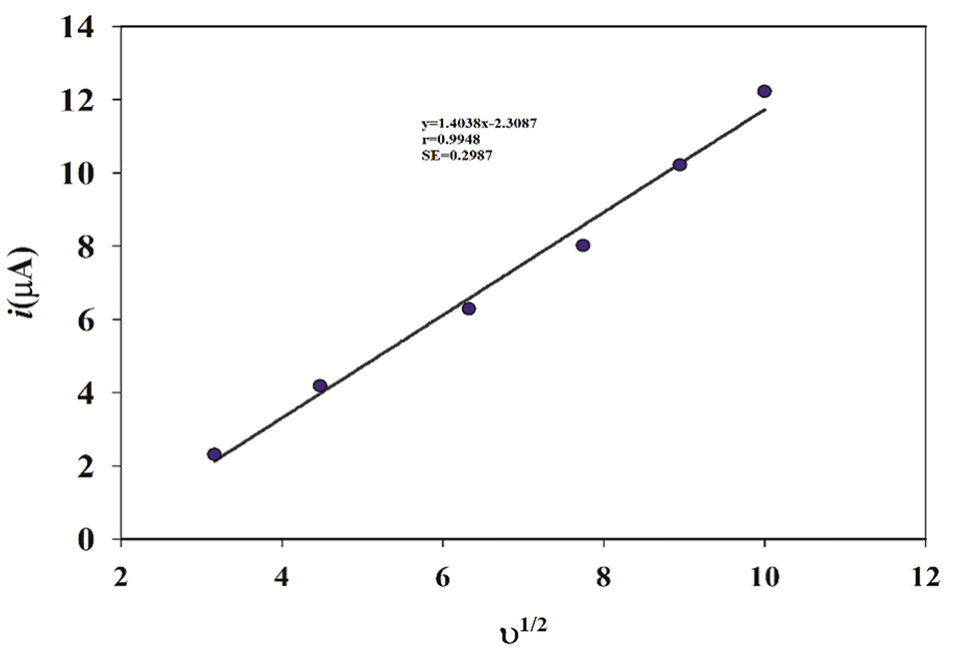
A plot of the anodic peak current (Ip) of PAR as a function of the square root of scan rate (ʋ^1/2^)

The impact of the scanning rate was studied on the anodic oxidation peak of PAR using CV. The CV curves of 0.1mM PAR on the Ni_0.75_Co_0.25_-MOFs/CPE were plotted in Fig. 8, with scanning rates ranging from 10.0 to 100.0 mV/s. Figure 9 demonstrates a clear linear relationship between the logarithm of peak current (I_P_) and the logarithm of scan rate (ʋ), the equation of regression log I_P_ = 0.6995 log ʋ − 6.3203 (*r* = 0.9976), showed that the slope is higher than the theoretical value of 0.50. This indicates that the anodic reaction at the electrode surface is a process controlled by diffusion, with some adsorption character. This regression equation I_P_=1.4038 ʋ^1/2^ − 2.3087 (*r* = 0.9948), demonstrated that there is a direct proportionality between, the peak current (I_P_) and the square root of the scan rate (ʋ^1/2^) within the range of scan rate from 10.0 to 100.0 mV/s, as shown in Fig. 10.

Moreover, as shown in Figure S4, during our study, the long-term stability of the developed sensor was assessed. The electrode was stored in the presence of air and measured every five days for two months to record the data regarding the change in the peak current values. The results show a loss of only 10.42% in the current value. Although bimetallic MOFs showed enhanced characteristics in terms of catalytic activity, they have a limitation of being commonly aggregated and difficulty controlling the pore structure. They lack the backbone to guard against structure deformation or aggregation. That shall direct our plans in future work to a template-assisted synthesis and controlled fabrication of “quasi-MOFs” [54].

#### Suggested mechanism of PAR electrochemical oxidation

The number of electrons involved in the reaction for an irreversible electrochemical process is calculated using the Laviron Eqs. [55, 56]

$$E=E \circ+2.303 R T / \alpha n F,[\log R \text { at } \mathrm{K} \circ / \alpha n F]+2.303 R T / \alpha n F(\log v)$$

**Scheme 1:**
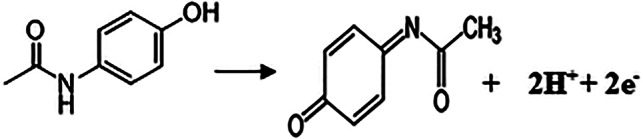
Electrochemical oxidation mechanism of PAR

All symbols have their standard meaning. For the system under study, using slope (0.0556), generated from linear plot of potential against log scan rate αn was calculated to be 1.032 for PAR at Ni_0.75_Co_0.25_-MOFs/CPE. Since an irreversible electron transfer α assumed to be 0.5 [56], therefore “n” value was calculated to be 2.064 PAR, which is consistent with 2 electron transfer processes involved in the oxidation of PAR at the modified electrode. Scheme 1 illustrates the possible electrochemical oxidation mechanism of PAR.

### Analytical performance of PAR

#### Calibration curve and detection limit

**Fig. 11:**
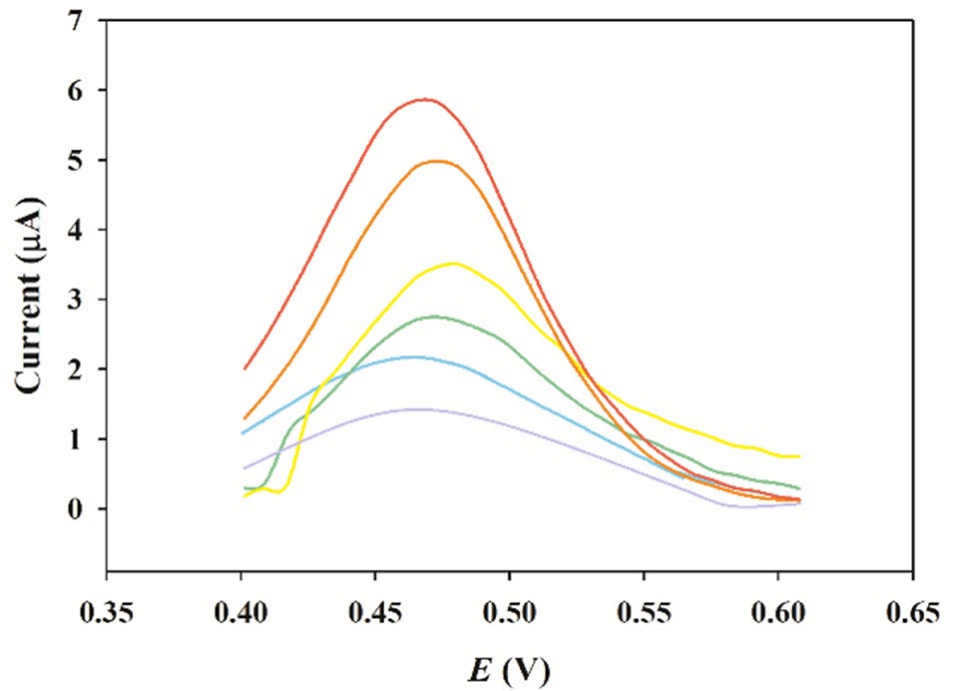
DPVs of different concentrations of PAR on the Ni_0.75_Co_0.25_-MOFs/CPE in 0.1 M PBS (pH = 7.4)

**Fig. 12:**
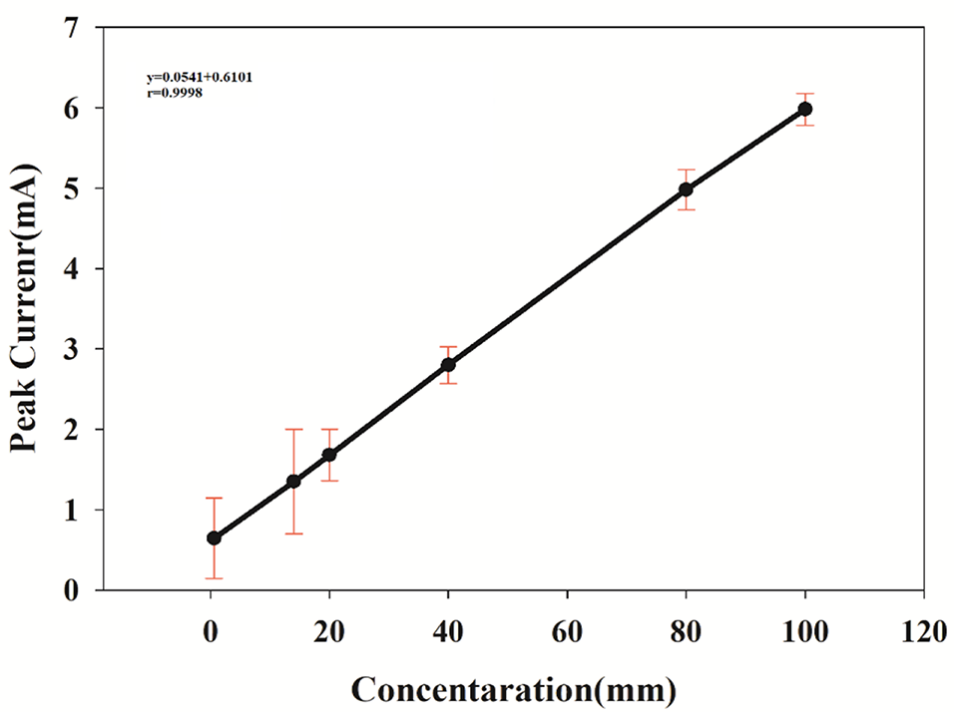
The peak current plot as a function of the concentration of PAR

The DPV method was used to measure the PAR peak current and was found to be linear within the range of 0.60–100.00 µM under the optimum electrochemical conditions as presented in Fig. 11, and Fig. 12 with the sensitivity of 0.0564 µA µΜ^−1^ cm^− 2^ and a low detection limit of 0.02 µM as mentioned in Table 1. A comparison between the detection limits/analytical range reported for PAR on various electrode systems [57–61] and that observed in the present work has been performed as shown in Table 2.

**Table 1:** Validation parameters obtained by the developed DPV method for determination of PAR in the presence of *p*-AP

Parameter	Paracetamol
LOD (µM) Linearity range (µM)	0.02 0.60–100.00
Slope	0.0541
Intercept	0.6101
Correlation coefficient (r)	0.9998
Accuracy (mean ± SD)	100.71 ± 1.30
Precision (%RSD) ^a^ (%RSD) ^b^	0.32 1.09

a Intraday precision [average of three different concentrations of three replicate each (n = 9) within the same day]

b Interday precision [average of three different concentrations of three replicate each (n = 9) repeated on three successive days]

**Table 2:** A comparison of analytical performance for PAR in electrodes modified with different materials

Modified electrodes	Method	Paracetamol		Reference
		Linear range (µM)	Detection limits (µM)	
2D Ni-MOFs/rGO/GCE	DPV	0.04–50.00	0.01	[57]
MoS_2_/Ni-MOF/SPGE	DPV	1.00–400.00	-	[58]
2D NiCu-CAT/GCE	DPV	5.00–190.00	-	[59]
HKUST-1/GCE	DPV	12.50–375.00	0.09	[60]
Cu(tpa)-GO/GCE	DPV	1.00–100.00	0.36	[61]
Ni_0.75_: Co_0.25_-MOFs/CPE	DPV	0.60–100.00	0.02	This work

### Method validation

**Fig. 13:**
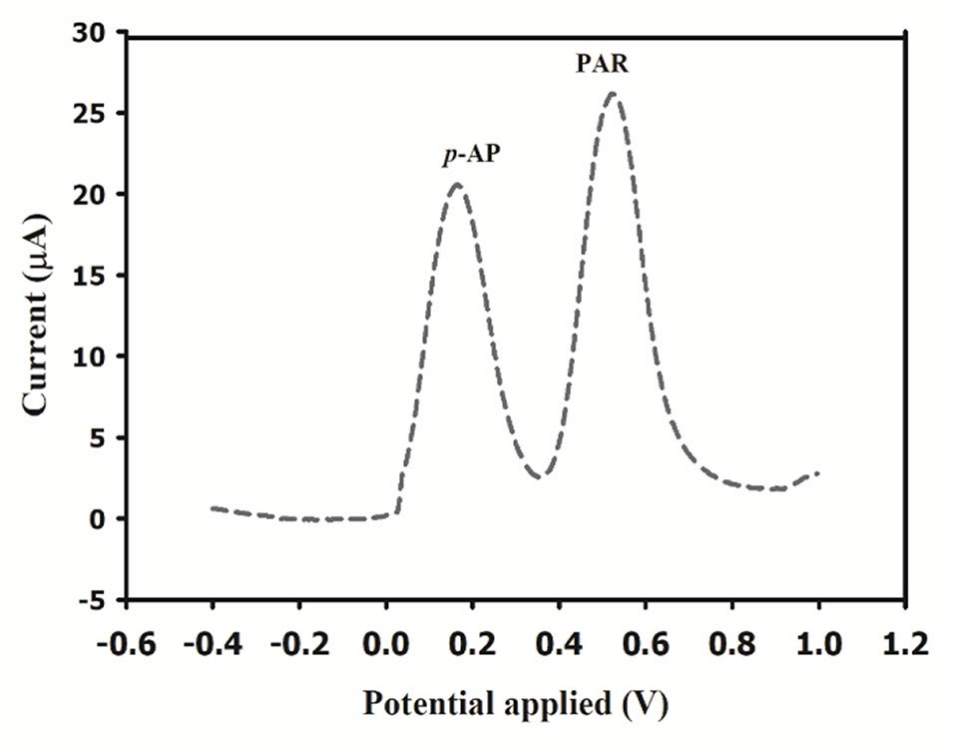
Differential pulse voltammograms of PAR and *p*-AP on the Ni_0.75_Co_0.25_-MOFs/CPE in 10 mM PBS (pH 7.4)

By examining three different concentration levels of PAR and calculating the percentage recovery, the suggested method was found to be accurate. The suggested method was precise based on the assessment of intra-day and inter-day precision, with % RSD values within the accepted range of less than 2%. The results of LOD, accuracy, and precision are shown in Table 1. As shown in Fig. 13, the method studied was also considered specific for its ability to efficiently detect PAR with uniform peak without interfering with its main impurity *p*-AP.

### Analytical applications

#### Analysis of PAR in the real samples

As our research was aimed at evaluating a new approach in practice, Ni_0.75_Co_0.25_-MOFs/CPE was used for PAR detection in its commercial tablet. As seen in Table 3, the recovery of PAR is from 98.77 to 99.81%, indicating the possible use of our electrochemical sensor to detect PAR in actual samples.

**Table 3:** Results of PAR analysis in real samples form by the developed DPV method

Product	Taken Concentration (µM)	Drug product Recovery %*±SD of the claimed amount
Panadol^®^ tablets each tablet is labeled to contain 500 mg of PAR Batch no.11,565,448	25.00	99.81 ± 0.74
	100.00	98.77 ± 0.70

#### Analysis of PAR in spiked human plasma

Tables 4 and 5 summarize the results of the accuracy and precision of the analysis of spiked human plasma samples by the suggested method. Our developed method was able to detect concentrations as low as 10^− 5^M, which is lower than the previously reported C_max_ value [62]. This confirms that our method is suitable for analyzing PAR in real human plasma samples.

**Table 4:** Analysis results of plasma samples spiked with different concentrations of PAR

Parameter	PAR spiked in plasma
Linearity range	1.00 × 10^− 5^ M-1.00 × 10^− 4^M
Slope	0.1032
Intercept	2 × 10^− 7^
Correlation coefficient (r)	0.9992
Accuracy	102.04 ± 0.49
Precision(%RSD) Intraday precision (Repeatability) Interday precision (Intermediate precision)	0.89 0.96

**Table 5:** Results of the proposed DPV method accuracy in spiked human plasma samples

Prepared concentration (M)	Found concentration(M)	^*^Recovery %
3.00 × 10^− 5^	3.05 × 10^− 5^	101.90
5.00 × 10^− 5^	5.13 × 10^− 5^	102.59
7.00 × 10^− 5^	7.13 × 10^− 5^	101.63
Mean ± SD		102.04 ± 0.49

### Statistical data analysis

The results obtained by Ni_0.75_Co_0.25_-MOFs/CPE were compared with those obtained by applying the official method for the determination of the PAR pure sample [27]. The t-test and F-value [63]were used to compare the mean and variance between the two methods where no significant difference in the results was found **(**Table 6**).**

**Table 6:** Statistical comparison of the results obtained by the proposed and official methods for the analysis of PAR in its pure form

Value	Proposed method	Official method ^[27]^
Mean	99.89	101.48
SD	1.50	0.89
Variance	2.25	0.79
n	6	5
Student’s t-test	2.074 (2.262)	
F value	2.848 (6.26)	

**Fig. 14:**
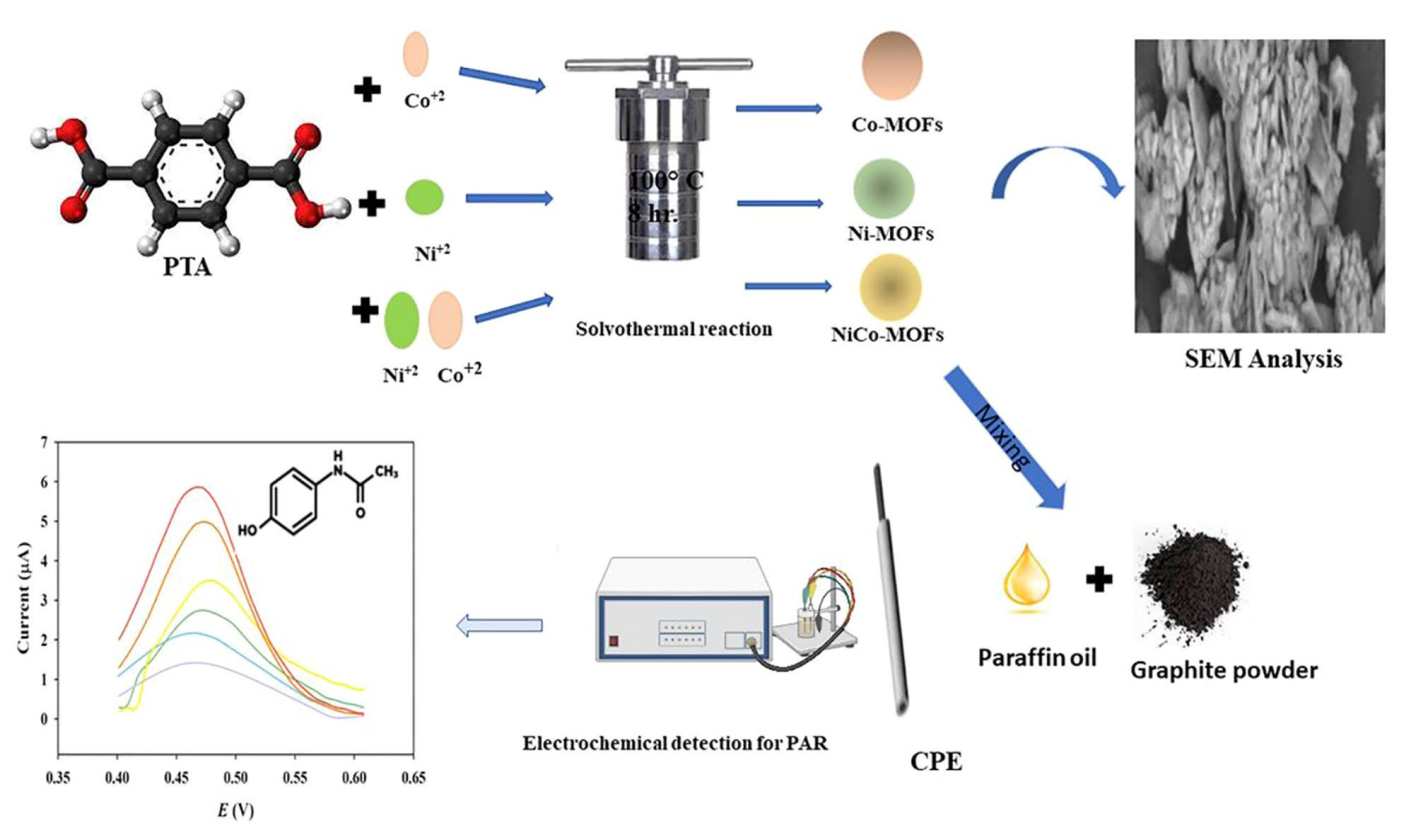
Schematic representation of monometallic and bimetallic MOFs synthesis and voltammetric measurements of PAR

The schematic representation of the electrochemical platform of MOFs synthesis and voltammetric measurements of PAR is summarized in Fig. 14.

## Conclusion

Bimetallic NiCo-MOFs with complex composition and structure show superior catalytic activity compared to their monometallic counterparts Ni-MOFs and Co-MOFs. An electrochemical sensing platform based on 2D Ni_0.75_Co_0.25_-MOFs/ CPE was successfully fabricated and employed in the detection of PAR in the presence of *p-*AP with a peak potential separation of 280.0 mV. Good electrochemical performance was attributed to the porous structure of MOFs, the good electrocatalytic activity, and the synergetic effect of Ni and Co. The constructed electrochemical sensor showed good selectivity and high sensitivity for PAR under optimized experimental conditions. The developed Ni_0.75_Co_0.25_-MOFs/CPE had a broad linearity range (0.60–100.00 µM) and a low limit of detection of 0.02 µM, this allows its use for pharmacokinetic studies in the future. In addition, the analysis of real samples and human plasma samples revealed the successful applicability of the developed sensor as a point of care in determining PAR. This work also provided a simple and general strategy for constructing MOFs-based electrochemical sensors.

### Electronic supplementary material

Below is the link to the electronic supplementary material.

Supplementary Material 1

## Data Availability

The datasets used and/or analysed during the current study are available from the corresponding author on reasonable request.
