# Supplementary material for: Bimetallic MOF-based electrochemical sensor for determination of paracetamol in spiked human plasma
Source: BMC Chem. 2024 Aug 8;18(1):148. doi: 10.1186/s13065-024-01247-7 (PMC11308493; doi:10.1186/s13065-024-01247-7)
Supplement: Supplementary file 1 — Supplementary Material 1 [file 13065_2024_1247_MOESM1_ESM.docx]

**Bimetallic MOF-Based Electrochemical Sensor for Determination of Paracetamol in Spiked Human Plasma**

Aya A. Mouhamed ^a^, Ahmed H. Nadim ^a*^, Amr M. Mahmoud ^a^, Nadia M. Mostafa ^a^, Basma M. Eltanany ^a^

^a^ Department of Pharmaceutical Analytical Chemistry, Faculty of Pharmacy, Cairo University, Cairo 11562, Egypt.

***Correspondence:**
Aya A. Mouhamed,

Faculty of Pharmacy - Cairo University

Kasr El-Aini St., Cairo 11562, Egypt

[aya.ahmed@pharma.cu.edu.eg](mailto:aya.ahmed@pharma.cu.edu.eg)

**Supplementary Figures**

**
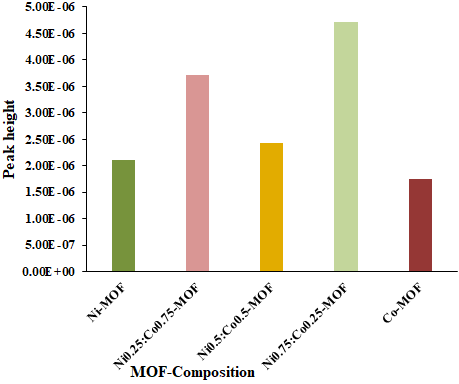
**

**Figure S1.** The peak current height of PAR oxidation at CPEs modified with different MOFs.


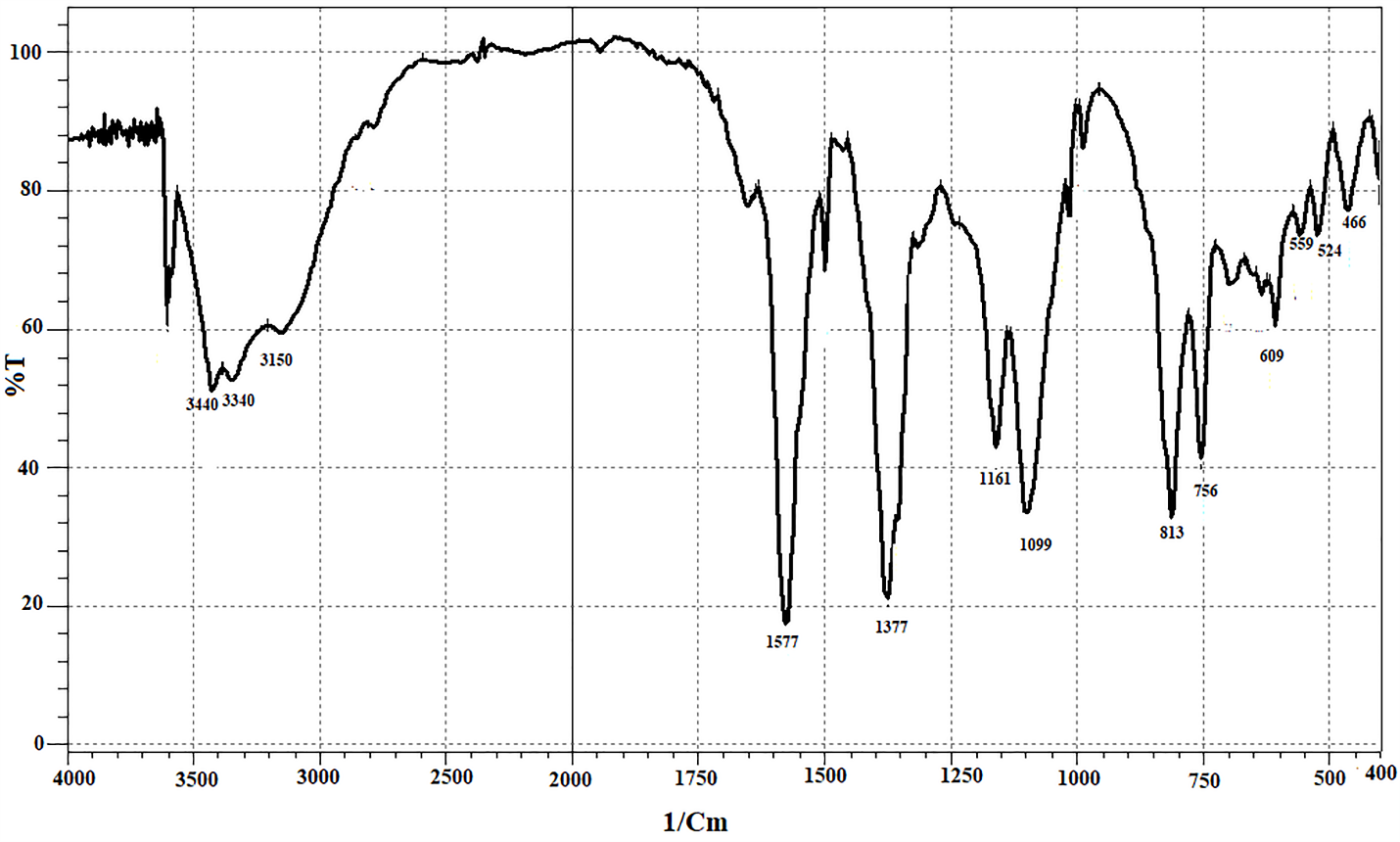


**Figure S2.** FTIR spectrum of the synthesized Ni_0.75_Co_0.25_-MOFs.


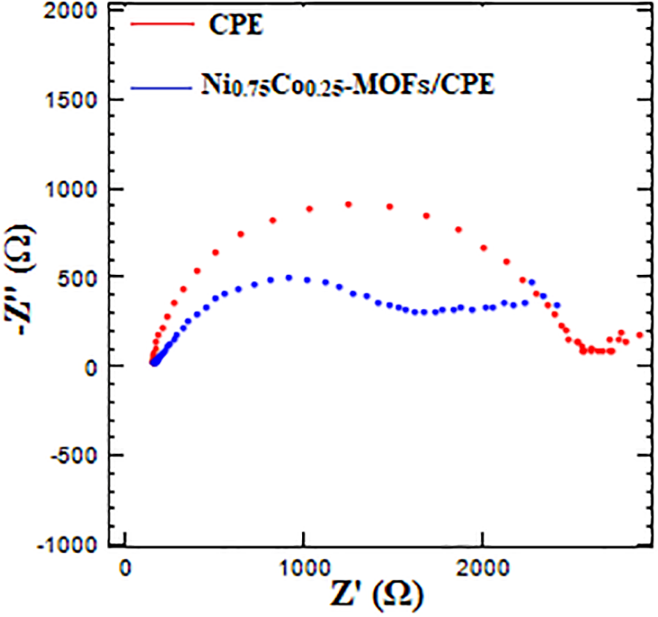


**Figure S3.** EIS of Ni_0.75_Co_0.25-_MOFs/ CPE, and bare CPE determined in a 10mM [Fe (CN)_6_]^-3^ /

[Fe (CN)_6_]^-4^ solution.


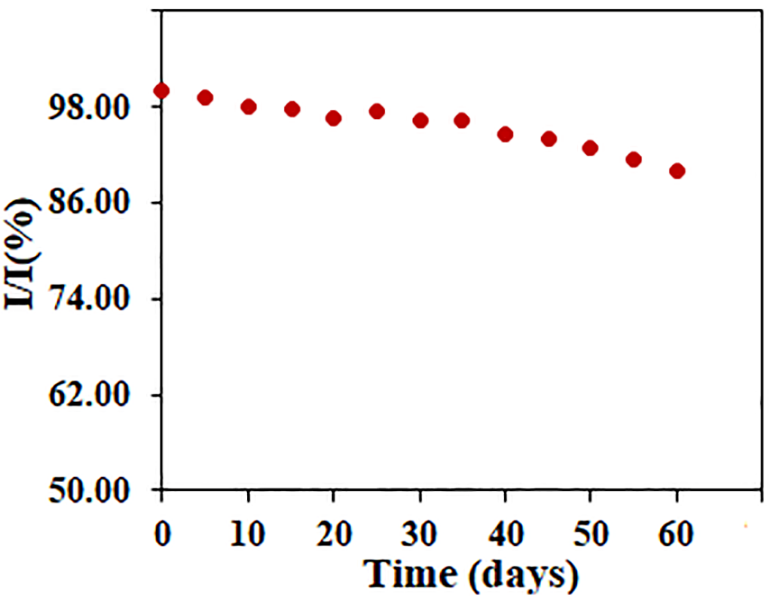


**Figure S4.** Long-term stability (1-60 days) of Ni_0.75_Co_0.25-_ MOFs /CPE electrode.
